# Supplementary material for: Heparin-based hydrogel scaffolding alters the transcriptomic profile and increases the chemoresistance of MDA-MB-231 triple-negative breast cancer cells
Source: Biomater Sci. 2020 Feb 13;8(10):2786–96. doi: 10.1039/c9bm01481k (PMC7497406; doi:10.1039/c9bm01481k)
Supplement: Supplementary file 2 [file BM-008-C9BM01481K-s002.zip › Supplementary File 4/HP-BhydrogelvControl/Pathways/my_analysis.Gsea.1545201079907/HALLMARK_REACTIVE_OXIGEN_SPECIES_PATHWAY.html]

Details for gene set HALLMARK\_REACTIVE\_OXIGEN\_SPECIES\_PATHWAY[GSEA]

|  || Dataset | expr.class.cls#HYD\_versus\_CONTROL.class.cls#HYD\_versus\_CONTROL\_repos |
| Phenotype | class.cls#HYD\_versus\_CONTROL\_repos |
| Upregulated in class | CONTROL |
| GeneSet | HALLMARK\_REACTIVE\_OXIGEN\_SPECIES\_PATHWAY |
| Enrichment Score (ES) | -0.44682056 |
| Normalized Enrichment Score (NES) | -1.6636313 |
| Nominal p-value | 0.009090909 |
| FDR q-value | 0.008763526 |
| FWER p-Value | 0.029 |
Table: GSEA Results Summary

  

Fig 1: Enrichment plot: HALLMARK\_REACTIVE\_OXIGEN\_SPECIES\_PATHWAY      
 Profile of the Running ES Score & Positions of GeneSet Members on the Rank Ordered List

  

| PROBE | DESCRIPTION (from dataset) | GENE SYMBOL | GENE\_TITLE | RANK IN GENE LIST | RANK METRIC SCORE | RUNNING ES | CORE ENRICHMENT || 1 | ABCC1 | na |  |  | 1714 | 1.485 | -0.0526 | No |
| 2 | SBNO2 | na |  |  | 1755 | 1.468 | -0.0182 | No |
| 3 | TXNRD2 | na |  |  | 1817 | 1.436 | 0.0143 | No |
| 4 | STK25 | na |  |  | 1846 | 1.424 | 0.0482 | No |
| 5 | SCAF4 | na |  |  | 2031 | 1.350 | 0.0722 | No |
| 6 | HHEX | na |  |  | 2388 | 1.213 | 0.0837 | No |
| 7 | LSP1 | na |  |  | 3350 | 0.953 | 0.0573 | No |
| 8 | ERCC2 | na |  |  | 3628 | 0.886 | 0.0648 | No |
| 9 | HMOX2 | na |  |  | 5440 | 0.562 | -0.0158 | No |
| 10 | EGLN2 | na |  |  | 6460 | 0.422 | -0.0585 | No |
| 11 | CDKN2D | na |  |  | 8255 | 0.224 | -0.1466 | No |
| 12 | GCLC | na |  |  | 9105 | 0.140 | -0.1874 | No |
| 13 | OXSR1 | na |  |  | 10410 | 0.006 | -0.2553 | No |
| 14 | SRXN1 | na |  |  | 11012 | -0.067 | -0.2850 | No |
| 15 | JUNB | na |  |  | 11042 | -0.071 | -0.2848 | No |
| 16 | GCLM | na |  |  | 11055 | -0.073 | -0.2836 | No |
| 17 | MBP | na |  |  | 11405 | -0.102 | -0.2993 | No |
| 18 | PFKP | na |  |  | 11433 | -0.105 | -0.2981 | No |
| 19 | GLRX2 | na |  |  | 11506 | -0.112 | -0.2991 | No |
| 20 | G6PD | na |  |  | 12043 | -0.179 | -0.3226 | No |
| 21 | FES | na |  |  | 12766 | -0.254 | -0.3540 | No |
| 22 | SOD2 | na |  |  | 13651 | -0.378 | -0.3907 | No |
| 23 | FTL | na |  |  | 14217 | -0.446 | -0.4091 | No |
| 24 | GPX4 | na |  |  | 14284 | -0.460 | -0.4012 | No |
| 25 | TXNRD1 | na |  |  | 14318 | -0.467 | -0.3913 | No |
| 26 | GPX3 | na |  |  | 14790 | -0.536 | -0.4025 | No |
| 27 | NQO1 | na |  |  | 14968 | -0.557 | -0.3980 | No |
| 28 | TXN | na |  |  | 15137 | -0.587 | -0.3921 | No |
| 29 | MSRA | na |  |  | 15361 | -0.628 | -0.3882 | No |
| 30 | GSR | na |  |  | 15538 | -0.659 | -0.3810 | No |
| 31 | PRDX1 | na |  |  | 16800 | -0.928 | -0.4238 | Yes |
| 32 | NDUFA6 | na |  |  | 16977 | -0.979 | -0.4086 | Yes |
| 33 | PRDX2 | na |  |  | 17621 | -1.207 | -0.4122 | Yes |
| 34 | NDUFS2 | na |  |  | 17809 | -1.291 | -0.3899 | Yes |
| 35 | PRDX4 | na |  |  | 17878 | -1.323 | -0.3606 | Yes |
| 36 | PRNP | na |  |  | 18243 | -1.523 | -0.3417 | Yes |
| 37 | ATOX1 | na |  |  | 18354 | -1.605 | -0.3076 | Yes |
| 38 | PDLIM1 | na |  |  | 18443 | -1.666 | -0.2707 | Yes |
| 39 | GLRX | na |  |  | 18498 | -1.703 | -0.2312 | Yes |
| 40 | MGST1 | na |  |  | 18735 | -1.933 | -0.1955 | Yes |
| 41 | SOD1 | na |  |  | 18799 | -2.015 | -0.1487 | Yes |
| 42 | PRDX6 | na |  |  | 18878 | -2.137 | -0.0997 | Yes |
| 43 | NDUFB4 | na |  |  | 18890 | -2.151 | -0.0468 | Yes |
| 44 | CAT | na |  |  | 19052 | -2.529 | 0.0076 | Yes |
Table: GSEA details [plain text format]

  

Fig 2: HALLMARK\_REACTIVE\_OXIGEN\_SPECIES\_PATHWAY      
 Blue-Pink O' Gram in the Space of the Analyzed GeneSet

  

Fig 3: HALLMARK\_REACTIVE\_OXIGEN\_SPECIES\_PATHWAY: Random ES distribution      
 Gene set null distribution of ES for **HALLMARK\_REACTIVE\_OXIGEN\_SPECIES\_PATHWAY**
